# Imaging findings of an obturator hernia: A rare cause of small bowel obstruction

**DOI:** 10.1016/j.radcr.2026.07.093

**Published:** 2026-08-31

**Authors:** Anna Tarasova, Raphael Miller, Mariia Samofalova, Steven J. Esses

**Affiliations:** Department of Radiology, Maimonides Medical Center, 4802 10th Avenue, Brooklyn, NY 11219

**Keywords:** Obturator hernia, Small bowel obstruction, CT

## Abstract

Obturator hernias represent an uncommon yet clinically important etiology of small bowel obstruction, and their subtle, nonspecific presentation often delays timely diagnosis. We report the case of a 94-year-old woman who presented with nonspecific abdominal complaints and was initially found to have a nonpalpable, nonobstructing hernia that was mistakenly characterized as inguinal in origin on initial CT interpretation. She was managed for obstructing renal stones and discharged, only to return shortly thereafter with persistent symptoms; reimaging with CT identified an obstructing obturator hernia. This case illustrates an important diagnostic pitfall, underscoring the ease with which an obturator hernia may be mistaken for a more common groin hernia. Through discussion of this diagnostic challenge, this article aims to clarify the anatomic distinctions and key imaging features that differentiate obturator, femoral, and inguinal hernias. Improved diagnostic accuracy is essential in this frail patient population to enable prompt surgical management and to minimize associated morbidity and mortality.

## Introduction

Obturator hernias are rare, accounting for 0.05%-1.4% of all abdominal wall hernias, yet carry disproportionately high morbidity and mortality (up to 70% in acute incarceration) secondary to diagnostic delays [1]. Classically presenting in elderly, emaciated women with bowel obstruction, obturator hernias are nonpalpable due to their deep anatomical location relative to the pectineus muscle [2]. Physical exam findings indicative of obturator nerve compression, such as the Howship–Romberg (medial thigh pain) and Hannington–Kiff (absent adductor reflex) signs [3], as well as intermittent symptoms secondary to spontaneous reduction [4] can provide crucial diagnostic clues; however, these clinical features are inconsistently present. Consequently, early cross-sectional imaging is essential for definitive preoperative diagnosis and timely surgical intervention.

## Case presentation

**Fig. 1:**
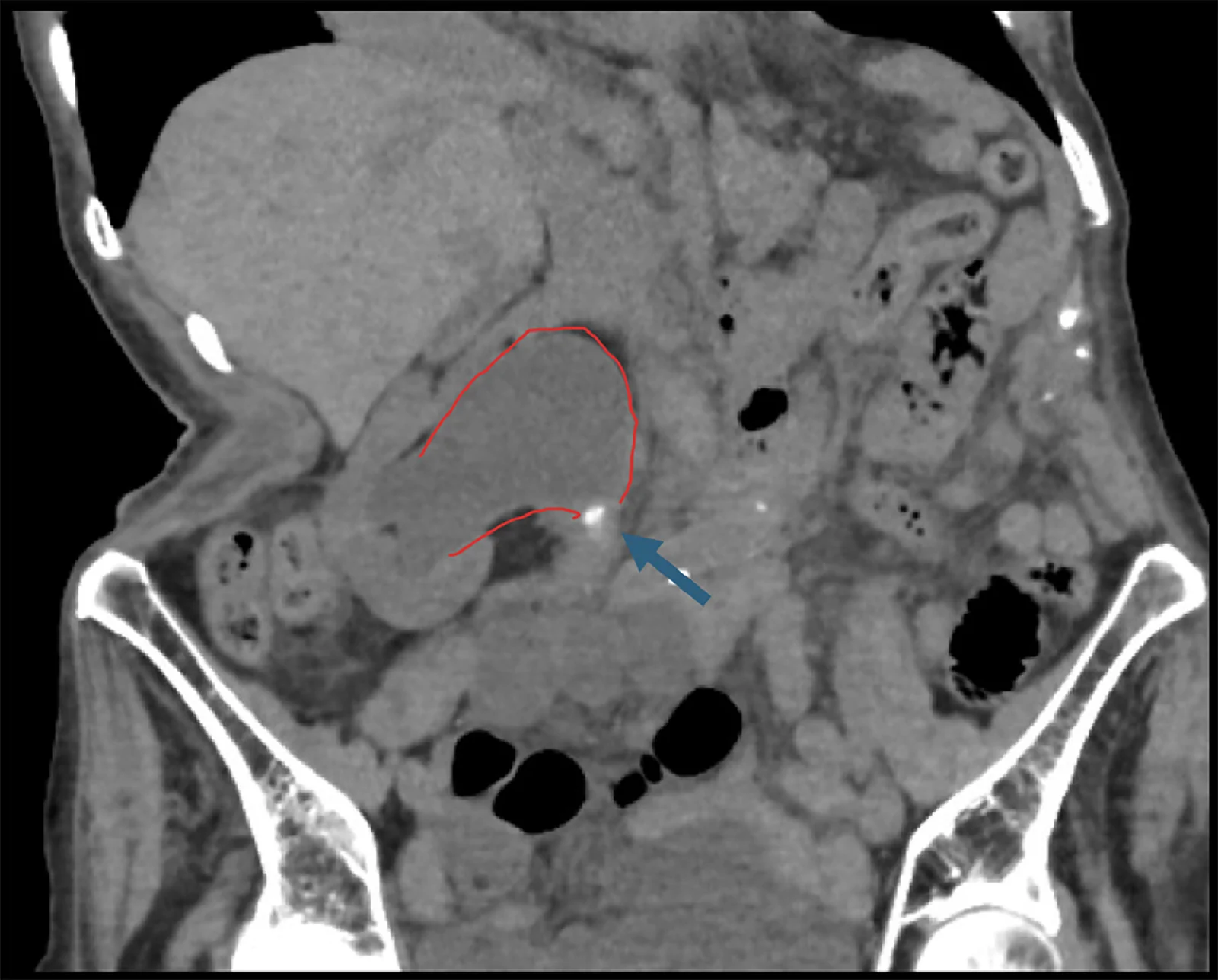
Coronal noncontrast CT image demonstrates sided hydronephrosis (red outline) secondary to a stone at the uteropelvic junction. Several nonobstructed loops of bowel are noted as well.

**Fig. 2:**
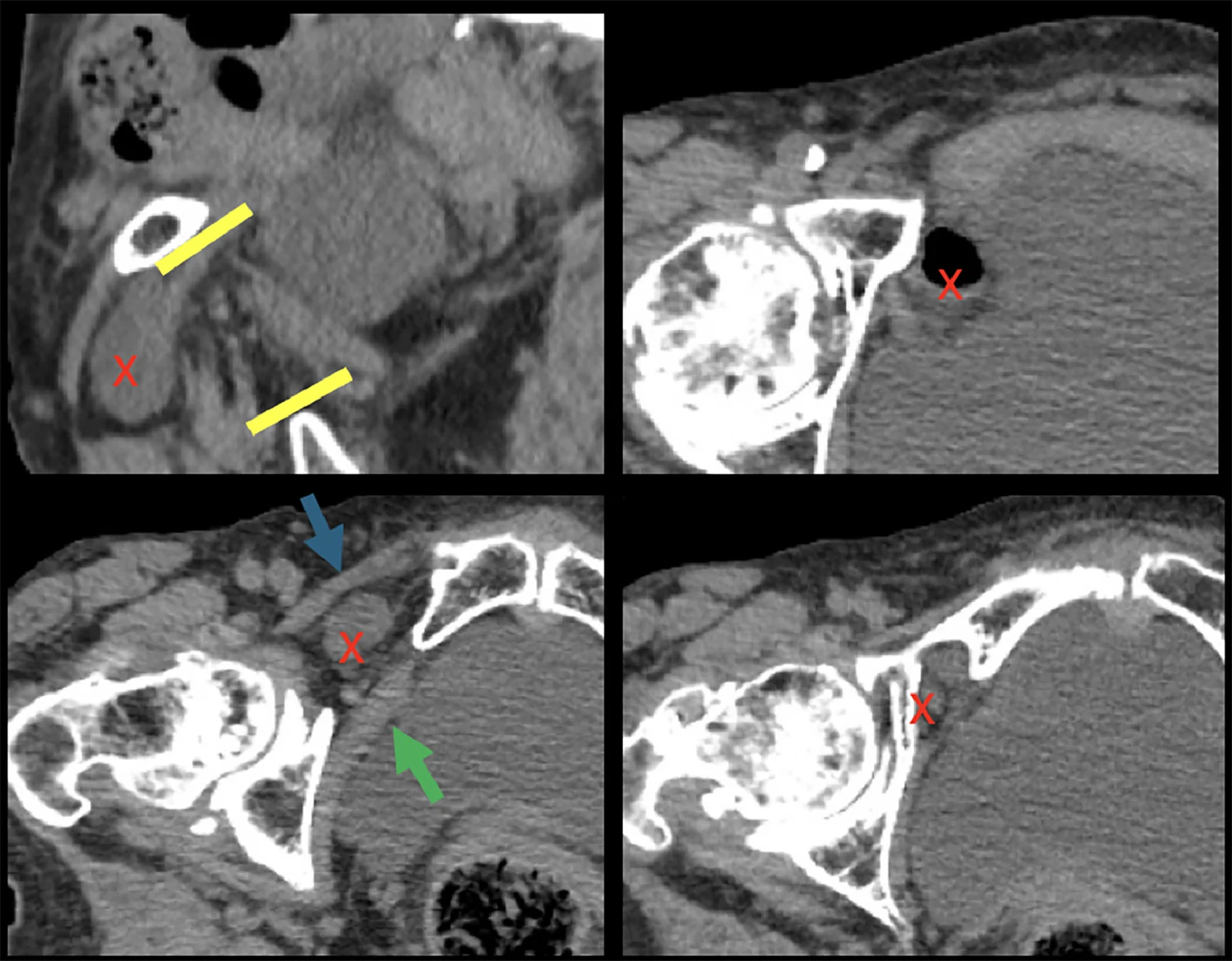
Clockwise from top left. Sagittal noncontrast CT image demonstrates a loop of bowel (red X) herniating through the right obturator foramen (yellow lines). Sequential axial CT images demonstrate a loop of bowel extending from right hemipelvis to the right obturator canal between the pectineus (blue arrow) and obturator (green arrow) muscles. The obturator canal is widened and measures approximately 2.2 cm. The left obturator canal is unremarkable (not shown).

A 94-year-old woman with a past medical history of hyperlipidemia, peripheral vascular disease, and recurrent nephrolithiasis presented to the emergency department with acute-onset right flank pain. The patient’s vitals and physical examination were unremarkable, and her laboratory studies revealed only a mild metabolic alkalosis. She was treated with analgesics and intravenous fluids. Given her history of nephrolithiasis, CT of the abdomen and pelvis was obtained, demonstrating a right ureteral stone and bilateral obstructing ureteropelvic junction stone (Fig. 1). A nonobstructing right hernia was also incidentally noted and reported as an inguinal hernia (Fig. 2).

On reassessment, the patient reported symptomatic improvement and requested discharge. She denied nausea or vomiting, and no palpable groin mass was identified on examination. As her creatinine remained normal and urinalysis was negative for infection, her obstructive uropathy was presumed to be chronic in nature, and she was discharged with strict return precautions. Additionally, outpatient follow-up was arranged with urology for her renal stones and with general surgery for her presumed inguinal hernia.

Three days later, she returned to the emergency department with persistent right flank pain and reduced oral intake. She appeared frail and ill-appearing, with coarse breath sounds noted on auscultation; the remainder of her examination and laboratory workup were unremarkable. She was again managed initially with intravenous fluids and analgesics. Urology was also consulted for cystoscopic management given her known obstructing renal stones.

Cystoscopy performed 2 days later under general anesthesia demonstrated right ureteropelvic obstruction secondary to a renal pelvic stone. The stone was pushed back and a double-J ureteral stent was placed, after which the patient reported resolution of her pain. She was discharged with outpatient follow-up again arranged with urology and general surgery.

The patient presented to the emergency department a third time the following day with dyspnea, nausea, vomiting, and abdominal distension. Her oxygen saturation was 93% and she was placed on supplemental oxygen. Respiratory rate, heart rate, and blood pressure were normal. Physical examination revealed bilateral rhonchi at the lung bases. Laboratory studies, including complete blood count, basic metabolic panel, and lactate, were unremarkable apart from a mild metabolic alkalosis.

**Fig. 3:**
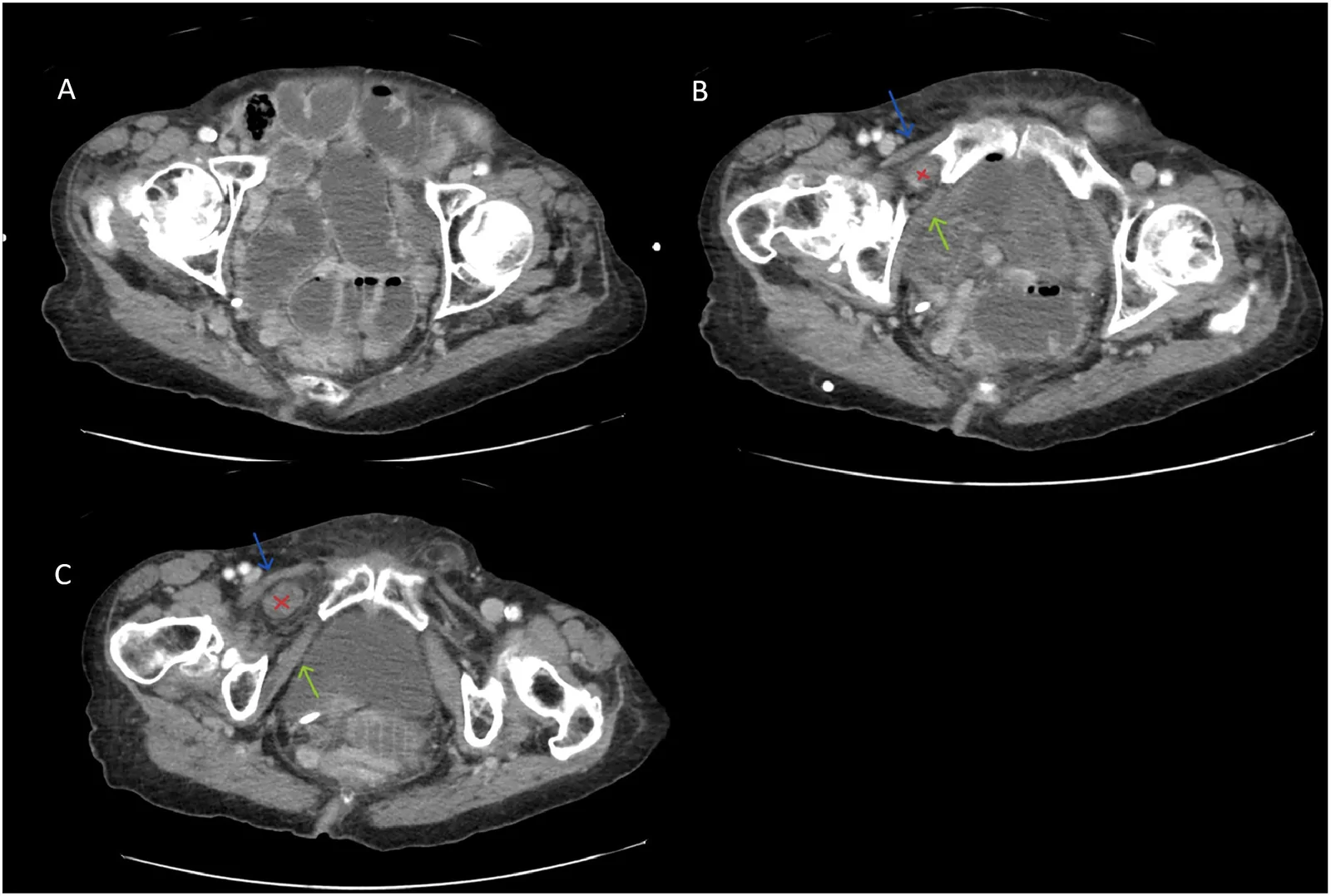
Sequential axial images from contrast-enhanced CT of the abdomen and pelvis demonstrate: (A) dilated loops of small bowel in the pelvis; (B) and (C) loop of small bowel (red X) interposed between the pectineus (blue arrow) and obturator musculature (green arrow). The obturator canal is widened and measures approximately 2.5 cm.

**Fig. 4:**
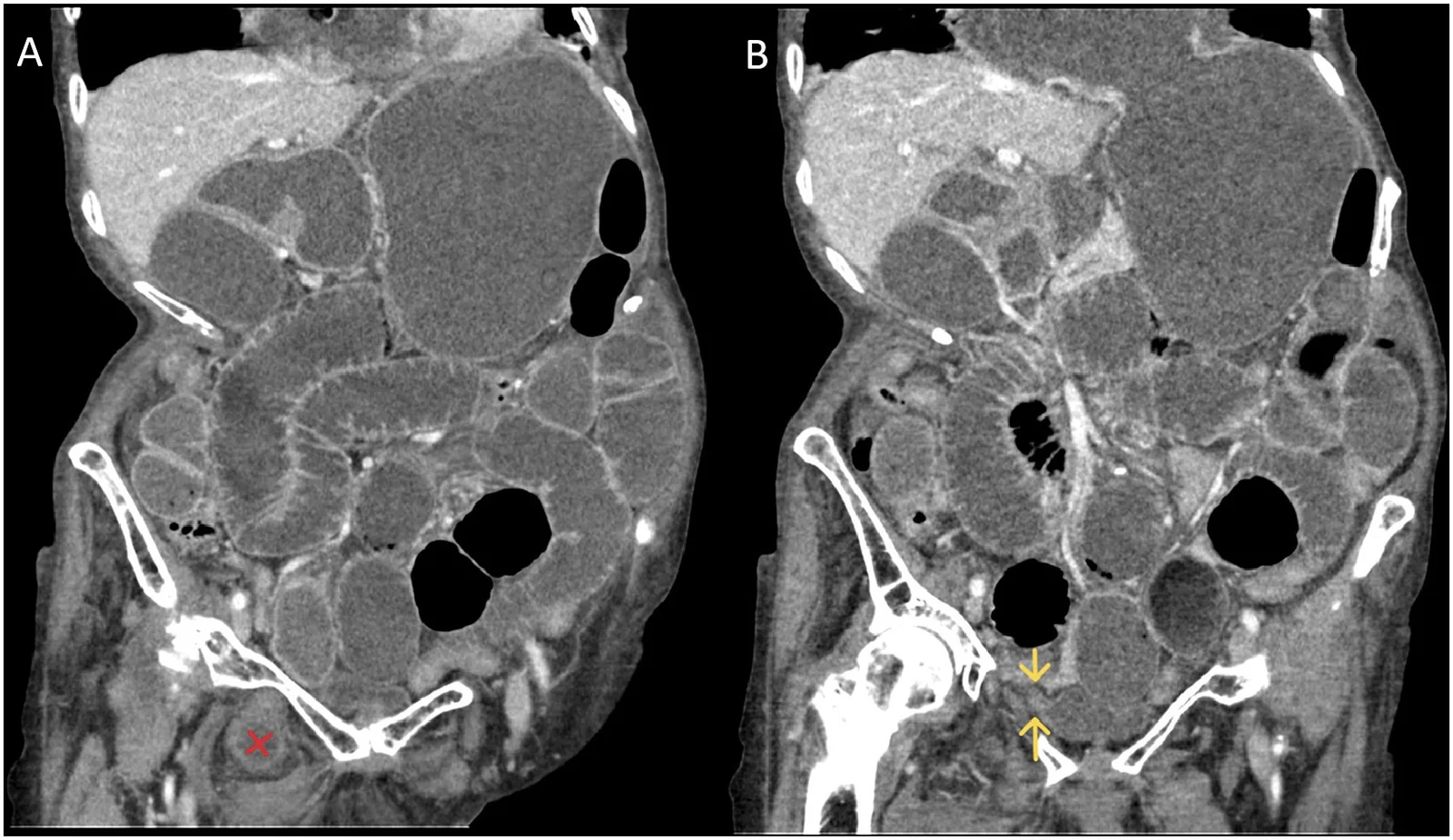
Sequential coronal images from contrast-enhanced CT of the abdomen and pelvis demonstrate: (A) diffusely dilated stomach and small bowel and herniated loop (red X); (B) transition point at the hernia neck (yellow arrows).

CT angiography of the chest was performed to evaluate for pulmonary embolism and pneumonia, and CT of the abdomen and pelvis was repeated to assess for intra-abdominal pathology. Imaging demonstrated a posterior right upper lobe consolidation and a small bowel obstruction with a transition point at the right obturator hernia (Figs. 3 and 4). A nasogastric tube was placed by the general surgery service, and the patient was started on broad-spectrum antibiotics and intravenous fluids and made NPO.

Later that day, she went to the OR for exploratory laparotomy. A strangulated loop of bowel was visualized herniating through the right obturator canal. Attempts at finger reduction were unsuccessful and the involved small bowel was resected. Postoperatively, she was kept on mechanical ventilation until postoperative day 3 due to aspiration pneumonia. She was ultimately discharged 1 week following surgery.

## Discussion

An obturator hernia develops through the obturator canal, a narrow defect located superiorly within the obturator foramen that is bounded by the obturator membrane and traversed by the obturator neurovascular bundle [5]. Although the herniated sac predominantly contains preperitoneal fat or small bowel, atypical contents such as the colon, appendix, greater omentum, Meckel’s diverticulum, urinary bladder [6], ovary, or fallopian tube have been described [7]. Anatomically, herniated viscera typically track deep to the pectineus muscle and anterior to the obturator musculature [8]. Given the unyielding boundaries of the canal, strangulation occurs frequently, leading to intestinal ischemia in up to 30% of cases [9].

Obturator hernias predominantly affect elderly, emaciated women, with a female-to-male ratio of approximately 6:1. Risk factors include a broader pelvis, a more triangular obturator canal, loss of protective preperitoneal fat, multiparity, increased intra-abdominal pressure, and the presence of multiple comorbidities [10].

CT imaging represents the definitive diagnostic modality for obturator hernias, providing superior visualization of the herniated sac and associated complications such as small bowel obstruction, intestinal ischemia, or perforation. By contrast, plain radiography may demonstrate nonspecific features of visceral obstruction or pneumoperitoneum in advanced cases [11]. Although ultrasound can occasionally aid in diagnosis, especially in elderly, thin females, its sensitivity is limited by operator subjectivity, suboptimal visualization of small or early-stage defects, and potential diagnostic confusion with femoral hernias [12].

**Fig. 5:**
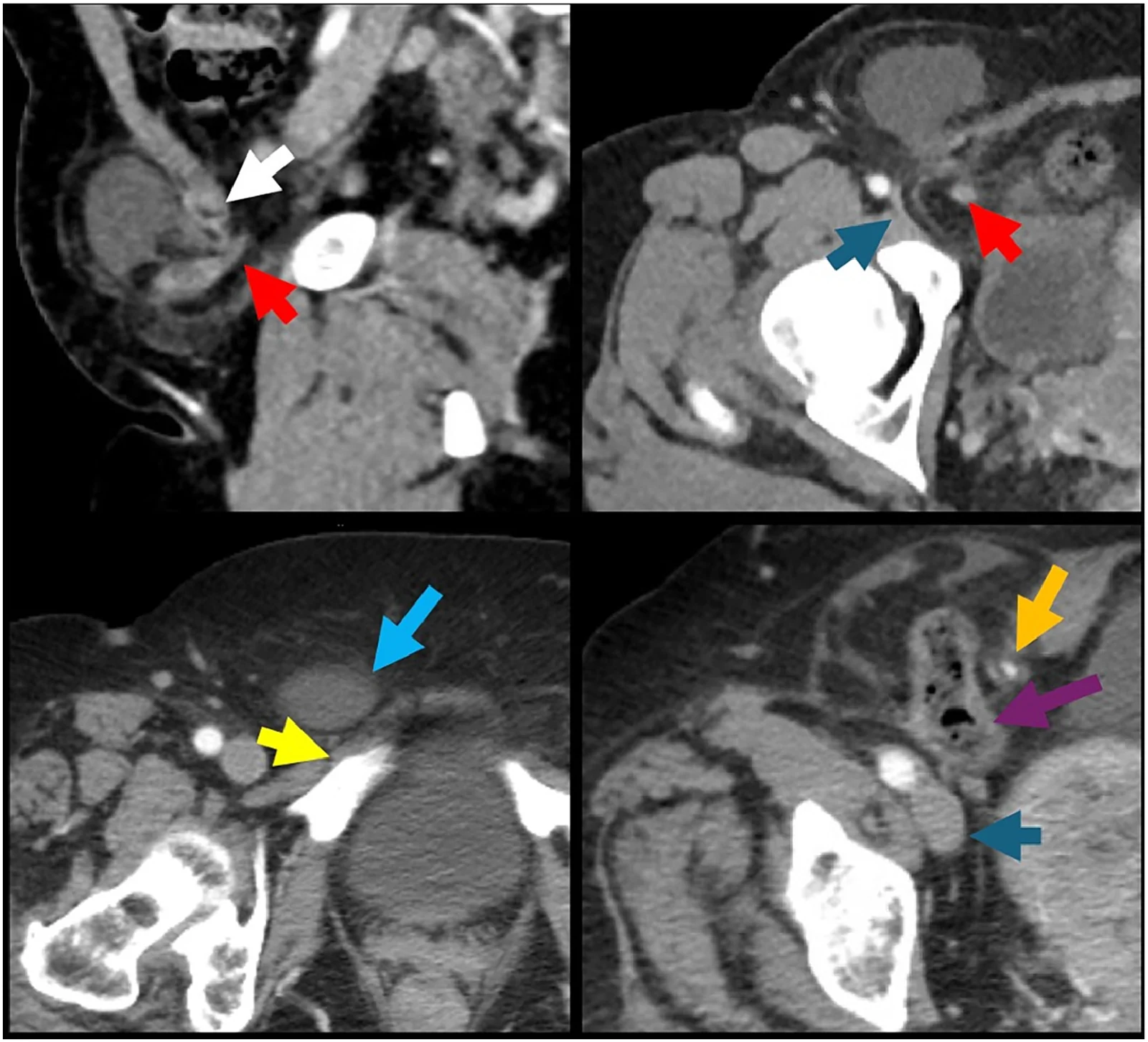
Clockwise from top left: Sagittal CT image demonstrates herniation of the appendix (red arrows) through a femoral hernia below the abdominal wall (white arrow). Axial CT image of the same patient showing herniation of the appendix (red arrow) through a femoral hernia medial to the femoral vein, which is compressed (blue arrow). Axial CT image of a different patient demonstrating herniation of a bowel loop (purple arrow) through an inguinal hernia lateral to the inferior epigastric vessels (orange arrow); there is no compression of the femoral vein at the level of the hernia neck (blue arrow). Axial CT image of the same patient demonstrates the hernia sac (light blue arrow) extending medial to the pubic tubercle (yellow arrow).

Accurate anatomic differentiation of various hernias is important. Femoral hernias typically exert mass effect on the femoral vein, whereas inguinal hernias characteristically extend medial to the pubic tubercle [13] (Fig. 5). Direct inguinal hernias project medial to the inferior epigastric vessels, whereas indirect inguinal hernias arise lateral to these vessels [14]. Finally, obturator hernias follow a distinctly anteroinferior course within the rigid confines of the obturator canal.

Although strangulation and high-grade bowel obstruction necessitated open exploratory laparotomy in this patient, laparoscopic repair is increasingly preferred when prompt diagnosis permits. Relative to open intervention, laparoscopic surgery offers reduced length of hospital stay, fewer complications, and decreased short-term mortality [15].

## Conclusion

Obturator hernia is a rare but life-threatening cause of bowel obstruction, predominantly affecting elderly, thin women. Its nonspecific presentation and deep anatomic location often delay diagnosis, and as illustrated by this case, and it may be mistaken for a more common groin hernia such as an inguinal hernia. CT is the diagnostic modality of choice and plays a critical role in early, accurate detection. Early recognition facilitates timely intervention, improves outcomes, and may allow for minimally invasive surgical management.

## Patient consent

Written informed consent for publication was provided by the discussed patient.
